# Reproducible research by design: design principles for low-code/no-code AutoML tools

**DOI:** 10.3389/frma.2026.1967319

**Published:** 2026-10-16

**Authors:** Armin Haberl, Stefan Thalmann

**Affiliations:** Center for Data Science in Business and Society (BANDAS), University of Graz, Graz, Austria

**Keywords:** AutoML, design principles, low-code/no-code, machine learning, reproducibility

## Abstract

Reproducibility is a major challenge for responsible machine learning (ML) research, especially with low-code/no-code automated ML (LCNC AutoML) tools making ML methods accessible to researchers outside data science. Existing approaches to support research reproducibility are difficult to apply to LCNC AutoML due to a lack of shareable code, limited tool transparency, and varying levels of ML expertise among users. To inform the design of LCNC AutoML tools that support reproducible research, this design science study proposes a set of design principles utilizing the theoretical lens of Technology Affordances and Constraints Theory. We first identified design requirements based on user studies with six researchers who implemented and reproduced LCNC AutoML projects. This was followed by a technical review of eight LCNC AutoML tools to identify existing design features in relation to the identified design requirements. Through mapping design features to design requirements, a set of design principles emerged: (1) GUI Customizability, (2) Standardized Reporting, (3) Interoperability, and (4) Adaptive Guidance. Together, these principles provide actionable design guidance for developing LCNC AutoML tools that support the documentation and reproducibility of ML research.

## 1 Introduction

Low-code/no-code (LCNC) development becomes more important in practice and research (Binzer et al., 2024) and in the field of machine learning (ML). Automated ML (AutoML) tools have made ML not only more accessible to practitioners but also to researchers without extensive ML expertise (Haberl and Thalmann, 2025). This accessibility is achieved by automating key stages of the ML pipeline (He et al., 2021) and offering intuitive graphical user interfaces (GUI) for LCNC development (Sundberg and Holmström, 2023). The resulting democratization of ML has led to the growing adoption of LCNC AutoML tools across disciplines beyond computer and data science (Scriven et al., 2023) and holds potential to transform how ML is applied in research.

While LCNC AutoML tools are user-friendly and capable of producing high-performing ML models (Paladino et al., 2023), they often lack explainability and transparency regarding their internal algorithms and decision processes (Sun et al., 2023; Xin et al., 2021). These limitations are particularly problematic in research, where transparent documentation of methodology is essential to ensure the reproducibility and trustworthiness of findings. Reproducibility challenges due to insufficient documentation are already recognized in ML research (Gundersen and Kjensmo, 2018), with particularly low documentation standards for ML methods in Information Systems (IS) research (Koenigstorfer et al., 2024a). As IS researchers increasingly develop and utilize ML models (Padmanabhan et al., 2022), promoting ML reproducibility can help foster more transparent and responsible IS research practices.

Several approaches have been proposed to support the reproducibility of ML research, including reproducibility guidelines, containerization of ML experiments, and awareness initiatives (Semmelrock et al., 2025). Although these approaches are promising for traditional ML research in computer science, their applicability to research involving LCNC AutoML remains limited. The absence of shareable code (Haberl and Thalmann, 2025), the limited transparency of underlying ML implementations (Sun et al., 2023), and the varying level of ML expertise among users (Pletzl et al., 2024; Sun et al., 2023) remain open issues. While recent studies have introduced promising methods to promote explainable (Zöller et al., 2023) and fairness-aware (Weerts et al., 2024) AutoML research, specific design guidance for AutoML tools that support research documentation or reproducibility are still missing. Previous studies on AutoML reproducibility also mainly focus on identifying reproducibility issues with current AutoML tools (Haberl and Thalmann, 2025; Pletzl et al., 2024) without providing actionable guidance to address these issues with improved tool design.

Addressing this research gap is important, as LCNC AutoML tools are often used in critical domains such as medicine (Castro et al., 2026), where the reliability and integrity of research findings are essential. Researchers who use LCNC AutoML are frequently not computer or data scientists and can lack the technical expertise required for comprehensive technical documentation (Haberl and Thalmann, 2025; Pletzl et al., 2024). Thus, developing LCNC AutoML tools that can support reproducible research by design could help to improve documentation practices and ultimately strengthen the reliability and integrity of research findings in various domains.

Design science research is suitable for addressing research problems that aim to develop and evaluate design knowledge (Hevner et al., 2004). This study therefore adopts a design science research (DSR) approach to identify design requirements and corresponding design features and to formulate four design principles for LCNC AutoML tools. The following research question guides this study: what design principles can guide the development of LCNC AutoML tools that can support reproducible research?

Drawing on insights from six AutoML implementation cases and a technical review of eight AutoML tools, we identified four design principles: (1) GUI Customizability, (2) Standardized Reporting, (3) Interoperability, and (4) Adaptive Guidance. Together, these principles offer actionable directions for developing LCNC AutoML tools that can support research reproducibility.

## 2 Background

LCNC development tools have been proposed to create digital solutions without the dependence on professional developers (Binzer et al., 2024). Previous research has highlighted the potential and challenges of using LCNC to develop artificial intelligence (AI) solutions in an organizational setting (Kandaurova et al., 2024). These LCNC tools can democratize AI and ML in organizations and bridge the gap between business and technology experts (Sundberg and Holmström, 2023). While the potential of LCNC AI tools in organizations is already broadly investigated, their impact within research settings remains relatively unexplored (Haberl and Thalmann, 2025).

LCNC AutoML tools allow researchers with limited ML expertise to create high-performance ML models with minimal to no code (Paladino et al., 2023). These tools automate various parts of the ML pipelines, such as data preparation, feature engineering, model generation, and model evaluation (He et al., 2021). While the extent of automation differs between tools, they can generally accelerate ML research and improve its efficiency (Karmaker et al., 2022). Especially, the introduction of LCNC user interfaces with drag and drop features enables the development of ML models without any coding experience (Raghavendran and Elragal, 2023). This has led to the use of LCNC AutoML tools in research fields outside computer or data science (Scriven et al., 2023).

The use of LCNC AutoML in research is becoming increasingly common, with researchers applying it in diverse fields such as medicine (Paladino et al., 2023), materials engineering (Conrad et al., 2022), and economics (Lazebnik et al., 2023). With LCNC AutoML tools, ML methods are easily accessible to researchers and offer advanced analytical perspectives for their research (Haberl and Thalmann, 2025). However, the automation and intuitive interfaces of LCNC AutoML tools also reduce the transparency of their internal mechanisms (Sun et al., 2023; Xin et al., 2021). This automation also reduces the agency of researchers and hides implementation details, which makes documentation and reproduction challenging (Haberl and Thalmann, 2025).

Reproducibility in ML research can be defined as “the ability of independent investigators to draw the same conclusions from an experiment by following the documentation shared by the original investigators” (Gundersen, 2021). (Gundersen 2021) defines four reproducibility types based on the documentation provided by the original investigators: (R1) Only a textual description is shared, (R2) a description and code are shared, (R3) a description and the dataset are shared, and (R4) the entire experiment including description, code, and data is shared. Providing more documentation tends to improve reproducibility while it can limit the generalizability of results (Gundersen et al., 2018). Despite ongoing efforts to promote reproducibility, the documentation practices in ML research are still largely insufficient in many disciplines (Koenigstorfer et al., 2024a).

Barriers to reproducibility in traditional ML research include incomplete research documentation, limited access to code and data, or the inherent randomness of many ML algorithms (Semmelrock et al., 2025). The need for accessing underlying datasets is often discussed using the FAIR (Findable, Accessible, Interoperable, and Reusable) principles for open data (Hey, 2022; Shanahan and Bezuidenhout, 2022; Wilkinson et al., 2016). Moreover, publication bias, data leakage during ML training, and insufficient computational resources can hinder reproducibility (Semmelrock et al., 2025). In addition to those barriers, LCNC AutoML has unique barriers, such as the lack of shareable code (Haberl and Thalmann, 2025), the hiding of implementation details (Sun et al., 2023), and limited ML expertise among researchers using these tools (Pletzl et al., 2024).

There are also several approaches to improve reproducibility in ML using technical approaches, reproducibility guidelines, or awareness initiatives (Semmelrock et al., 2025). A solution to provide computational reproducibility is the use of containers that bundle the different components and dependencies of ML experiments into a single executable package (Moreau et al., 2023). A perspective article by (Schubotz et al. 2022) also recommends the introduction of caching (saving intermediate results for later use) to make computationally expensive ML experiments easier to reproduce. Publishers also started to implement reproducibility checklists, which can lead to an overall increase in code and data sharing (Pineau et al., 2021). However, it is currently unclear to what degree the reproducibility approaches of traditional ML research are transferable to LCNC AutoML.

To investigate how to support reproducible research with LCNC AutoML tools, this study uses the theoretical lens of Technology Affordances and Constraints Theory (TACT) introduced by (Majchrzak and Markus 2012). This theory extends insights from affordance theory (see e.g., Gibson, 1977; Volkoff and Strong, 2017) and defines two main concepts: (1) technology affordances are goal-oriented action potentials that technology provides to individuals or organizations; (2) technology constraints are barriers that prevent individuals or organizations from achieving their goals when using technology. TACT has already been used in IS research to study AI in various contexts, such as deepfakes with generative AI (Lundberg and Mozelius, 2025), AI for professional services (Yang et al., 2024), or AI in public sector organizations (Maragno et al., 2023). However, the affordances and constraints of LCNC AutoML tools and their implications for research reproducibility have not yet been studied.

## 3 Materials and methods

Our main goal is to develop design principles for LCNC AutoML tools supporting research reproducibility. Following the eDSR methodology by (Tuunanen et al. 2024), the study consists of two main empirical components. First, implementation cases with researchers were conducted to identify design requirements. Second, a technical review of existing LCNC AutoML tools was conducted to assess the operationality of these requirements by identifying corresponding design features. The resulting requirements and features informed the formulation of the design principles. Sections 3.1 and 3.2 describe the two components in detail, and Figure 1 provides an overview of the research process.

**Figure 1:**
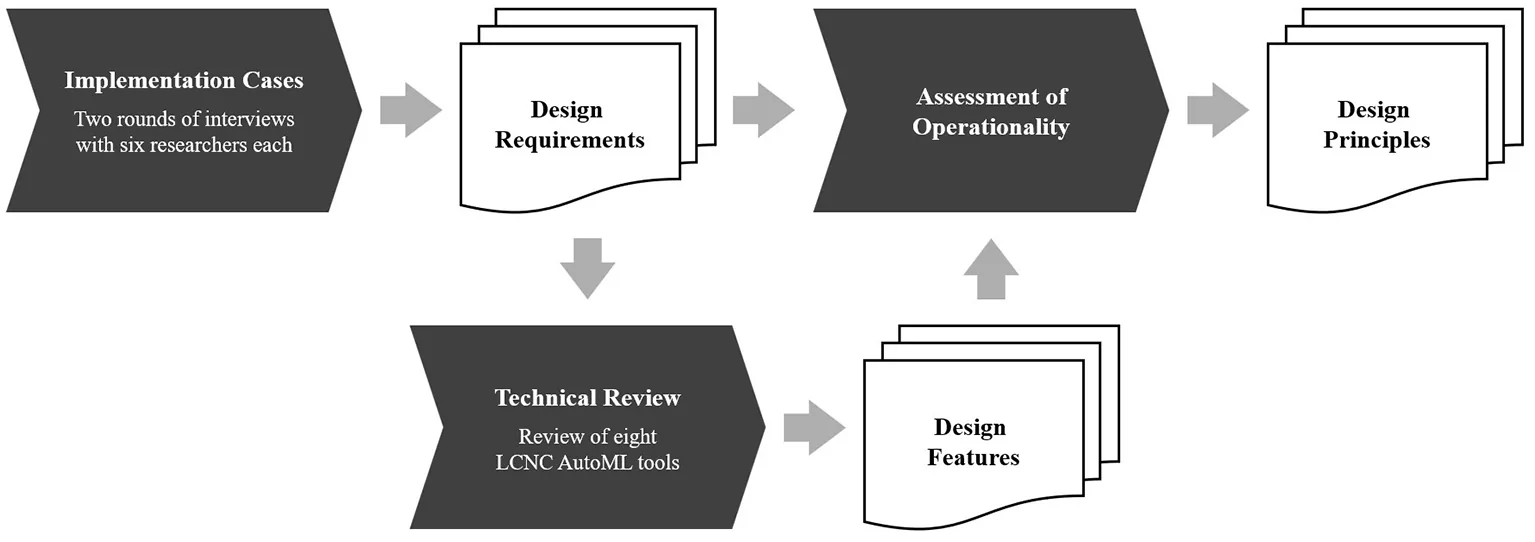
Overview of the research methodology.

### 3.1 Implementation cases

To identify design requirements for LCNC AutoML tools, we conducted six implementation cases where researchers implemented and reproduced AutoML projects between October 2024 and February 2025. The researchers recruited for the implementation cases were all located at an Austrian university and were experts in various research domains (see Table 1). None of the researchers were experts in data science or computer science.

**Table 1:** Overview of the participants in the implementation cases.

ID	Domain	AutoML Tool	Data Type	ML Task	Interview 1	Interview 2
**P1**	Psychology	KNIME	Tabular	Classification	52 min	30 min
**P2**	Law	KNIME	Tabular	Classification	50 min	25 min
**P3**	Business	KNIME	Tabular	Regression	66 min	51 min
**P4**	Sociology	KNIME	Tabular	Regression	48 min	24 min
**P5**	Business	Dataiku	Image	Classification	66 min	22 min
**P6**	Ethics	Dataiku	Tabular	Classification	36 min	26 min

Each researcher completed an AutoML project and then exchanged their project with another researcher for reproduction in pairs. The researchers selected datasets and ML tasks that aligned with their own research or personal interest but were still simple enough to be understood by the subsequently reproducing researchers. The datasets did not require expert knowledge in a specific research domain such as law or psychology to be understood but rather represented generally understandable data problems. We also introduced the researchers to a set of widely used AutoML tools described in (Egger 2022), from which four researchers selected KNIME^1^ and two researchers selected Dataiku^2^ as tools for their AutoML project. A detailed overview of the implementation cases, including the used tool, ML tasks, datasets, reproduction targets, and reproduction outcomes, can be found in Supplementary material. The researchers had to create research documentation of their AutoML project that was shared with the reproducing researchers. The reproduction attempts were carried out based on this research documentation and documented as well. To support documentation, we provided the researchers with documentation templates for both the implementation as well as the reproduction task, which can also be found in the Supplementary material.

Data of these implementation cases were collected through semi-structured interviews with the participating researchers. For each case, we conducted two interviews: one after the initial implementation and another after the reproduction attempt. The implementation interview contained questions about the initial research situation, familiarization with the tool, model creation and evaluation methods, documentation approach, and learning effects. The reproduction interview contained questions about the initial reproduction situation as well as the approach, success, and issues of the reproduction attempt. Both interview guidelines are available in the Supplementary material. Based on these guidelines, we conducted a total of 12 interviews, with transcripts automatically created using aTrain (Haberl et al., 2024) and manually checked for transcription errors.

We subsequently analyzed the interview data following an informed-inductive approach, specifically the theme analysis described in (Mayring 2014). In this approach, a main category system is defined based on an existing theoretical framework and the material is coded deductively based on those categories (Mayring, 2014). Within the categories, the material is then summarized and clustered into themes using an inductive approach (Mayring, 2014). The main category system in this study was derived from the reproducibility types introduced by (Gundersen 2021): Description, Code, Data, and Experiment.

The following example should clarify the analytical approach (see also Table 2). For example, within the category *Experiment*, we deductively identified the codes “Tool does not provide explanation of performance metrics,” “It requires statistics know-how to interpret model performance,” and “Performance metrics are difficult to understand for non-experts.” These codes were later clustered inductively into the theme *Only expert quality metrics*. The themes were then classified as affordances or constraints using the theoretical lens of TACT (Majchrzak and Markus, 2012). The theme *Only expert quality metrics* was classified as a constraint, as it represents a barrier that prevents researchers from adequately understanding and documenting ML outcomes. The final codebook outlining the entire coding scheme can be found in the Supplementary material.

**Table 2:** Illustrative examples of the analytical approach adapted from (Mayring 2014).

**Category based on (Gundersen 2021)**	Exemplary codes from material	Exemplary theme emerging from codes	Theme classification based on (Majchrzak and Markus 2012)
Description	•Tool interface can be documented using screenshots •Tool interactions can be documented using screencasts or videos •Screenshots of the tool interface are helpful during reproduction	Creating visual descriptions	Affordance
Code	•Imported workflows are incompatible with other tool features •Complete workflow with intermediate results cannot be shared with others •Tool does not allow users to export the ML workflow as code	Limited workflow interoperability	Constraint
Data	•Tool provides visualizations of dataset characteristics •Tool provides information about missing values in the dataset •Tool provides information about outliers in the dataset	Reporting data characteristics	Affordance
Experiment	•Tool does not provide explanation of performance metrics •It requires statistics know-how to interpret model performance •Performance metrics are difficult to understand for non-experts	Only expert quality metrics	Constraint

Based on the identified constraints, we finally identified design requirements for AutoML tools to support research reproducibility (see Table 3). We decided to base the design requirements on the identified constraints, as they represent the current barriers for the documentation and reproducibility of research conducted with LCNC AutoML tools. Thus, tackling these constraints represents a promising avenue to promote reproducible research with LCNC AutoML.

**Table 3:** Overview of identified affordances, constraints, and design requirements.

Type	Affordances	Constraints	Design requirements (DR)
Description	Creating visual descriptions	Fixed visual information	DR1: Personalizing the visual representation of workflows
	Creating individual reports	No predefined reporting	DR2: Creating predefined reports automatically
Code	Sharing workflows	Limited workflow interoperability	DR3: Easily accessing interoperable workflows
	Recreating and executing workflows	Missing workflow lineage	DR4: Documenting design decisions within workflows
Data	Reporting data characteristics	Missing data utility information	DR5: Assessing data utility and risks
	Reporting data processing	No standardized data processing	DR6: Importing and processing data in a standardized way
Experiment	Reducing stochastic variation	No reproducibility guidance	DR7: Understanding implications of settings on reproducibility
	Reporting model performance	Only expert quality metrics	DR8: Assessing the quality of AutoML outcomes
	Reporting hardware and software setup	Unknown hardware and software interdependencies	DR9: Understanding hardware and software interdependencies

The coding process was iterative, involving several revisions to the code system and themes. To support the reliability of our analysis, we applied a consensus validation approach (Cascio et al., 2019). Specifically, the interview data were coded by one author, and the coding results were subsequently reviewed and discussed jointly by both authors. Interpretation differences were resolved through discussion, and the coding scheme was iteratively refined and adapted until a shared understanding of categories, themes, and their definitions was achieved.

### 3.2 Technical review

To evaluate the operationality of the identified design requirements, we conducted a technical review in which we compared them with the design features of existing LCNC AutoML tools. Building on (Tuunanen et al. 2024), we define *operationality* as the capability of design requirements to be turned into concrete features within LCNC AutoML tools. Assessing the operationality of the design requirements thus provides a basis for prototypical instantiations that can subsequently be evaluated regarding their effects on reproducibility. We selected eight common LCNC AutoML tools (see Table 4) for citizen developers and researchers, as suggested by (Egger 2022). This set of tools was selected to provide a balanced sample of proprietary and open-source solutions, as well as a combination of locally installed and cloud-based systems. Evaluating the design requirements against additional AutoML tools also reduces the risk of overfitting them to the tools used in the implementation cases.

**Table 4:** Overview of the reviewed LCNC AutoML tools.

ID	Tool name	Version	Website
T1	Altair AI Studio	2026.0	https://docs.rapidminer.com
T2	Orange Data Mining	3.39.0	https://orangedatamining.com/docs/
T3	KNIME	5.8.0	https://docs.knime.com/
T4	WEKA	3.8	https://waikato.github.io/weka-wiki/
T5	SAS Viya	2025.03	https://support.sas.com/en/documentation.html
T6	BigML	Andromeda	https://support.bigml.com/
T7	Dataiku DSS	14.1.4	https://doc.dataiku.com/
T8	DataRobot	11.1	https://docs.datarobot.com/

The technical review was conducted in October 2025 and involved systematic hands-on experimentation within the tools as well as an in-depth analysis of their official documentation. Specifically, we first installed all local AutoML tools on a Dell Latitude 5530 laptop with 16 GB RAM and a CPU-integrated GPU (Intel UHD Graphics) running on Windows 11 Pro (64-bit). For the cloud-based systems, we created user accounts and activated the free trial versions for our investigation. The hands-on experimentation within the tools involved creating one complete ML workflow reaching from data import and model training to model evaluation and documentation. Specifically, we performed a regression task in which we tried to predict student performance based on prior academic metrics using a publicly available dataset. Alongside this hands-on experimentation, we recorded all the functionalities available in the tools that corresponded to the previously identified design requirements. Additionally, we also analyzed the official documentation offered by the tool providers to get a richer understanding of the functionalities we might have missed in our implemented ML workflow.

The initial data collection during the technical review was conducted by one of the authors and resulted in a concrete list of tool functionalities related to each design requirement. Through an inductive process, we further clustered the specific functionalities into higher-level design features. For example, functionalities such as “Export workflow, data and model information as text file,” “Data lineage / data transformation report for datasets,” and “AI explanations for every step in a workflow report” were clustered in the design feature “Automated Report Generation.” A more detailed overview of the technical review protocol, including a link to the used dataset for implementing the ML workflows, and concrete descriptions of the identified functionalities and design features can be found in the Supplementary material.

To support the reliability of the technical review results, we again used a consensus validation approach (Cascio et al., 2019), where any differences in interpretation were iteratively resolved through discussions among both authors. Through this iterative process, we identified concrete design features that addressed specific design requirements for research reproducibility. This demonstrates the operationality of the design requirements and provides a basis for subsequent prototypical implementations that could be evaluated for their effects on reproducibility. After mapping the identified design features to their corresponding design requirements and incorporating insights from the literature we formulated four design principles for LCNC AutoML tools supporting research reproducibility.

## 4 Results

In the following section, we first present the design requirements for future LCNC AutoML tools identified through the implementation cases. We then outline the results of the technical review of current LCNC AutoML tools, comparing their design features to the requirements identified through implementation cases. Finally, we propose four design principles that serve as an abstraction layer between design requirements and design features.

### 4.1 Identification of design requirements

The results in this section are based on both the interviews conducted after the initial implementation as well as after the reproduction attempts. The section is structured using the main category system used during the qualitative data analysis (see Section 3.1), which is based on the reproducibility types introduced by (Gundersen 2021): description, code, data, and experiment. For each reproducibility type, we introduce the affordances and constraints that inductively emerged from the coded material. Finally, we propose design requirements (DR) that address the constraints observed in current tools (see Table 3) and aim at improving documentation practices and reproducibility of research with LCNC AutoML.

#### 4.1.1 Description

To support the reproducibility of AutoML results, researchers must provide a description of their AutoML projects, such as procedures, implementations, or research outcomes.

*Affordance 1—Creating visual descriptions*: Our findings show that AutoML tools afford researchers to create visual descriptions of their AutoML projects. Researchers were able to take screenshots of important information within the tools and therefore provide implementation details of their AutoML projects for their reports. One researcher explained:

“Everything I changed [was documented] with screenshots, every task with screenshots.” (P5)

Furthermore, some researchers also created screencasts of the tools' GUI and thereby documented every action they performed in their AutoML project. Another researcher noted:

“If we focus purely on this methodical reproduction […] a short video is definitely the clearest option because then there is actually no room for interpretation.” (P6)

These results suggest that the LCNC interfaces of AutoML tools not only facilitate the implementation of AutoML projects but also support their documentation in a visualformat.

*Affordance 2—Creating individual reports:* AutoML tools also afford researchers to create individual reports of their AutoML projects using free-text descriptions. As AutoML tools offer highly automated workflows, researchers can typically configure and describe only a limited number of parameters. One researcher noted that they could postpone the documentation process since there were very few settings to record:

“I think that because it was so easy, it was also good to do it the way I did, documenting it afterwards.” (P2)

This limited number of configuration options also allows researchers to create more concise textual documentation of their AutoML projects. For example, one researcher noted that basic textual descriptions were enough to reproduce the methodology of other AutoML projects:

“Not every single click was documented, but it was totally sufficient for the context in which we were working.” (P1)

These findings suggest that the automated workflows and limited configuration options in AutoML tools simplify the creation of individual textual documentation andreports.

*Constraint 1—Fixed visual information:* The highly automated visual workflows of AutoML tools also lead to a constraint, where only fixed visual information is displayed, and other underlying implementation details remain hidden. This fixed visual information made it particularly difficult for researchers to provide justifications for other not displayed implementation details within their documentation. One researcher noted:

“And that depends on the tool again, but sometimes you don't really know what [implementation] they are using. I can't really justify that.” (P2)

In addition, the available visual information is often expressed in expert-level data science terminology, which makes it difficult for researchers to document the underlying processes of AutoML. For example, one researcher suggested that the tools should better explain the available visual information:

“If I say, I now have my parameter XY, and then I click on it somehow, and then there's a little pop-up window that explains to me what it is. That would certainly be quite good for understanding.” (P6)

These findings suggest that current AutoML tools do not provide sufficient flexibility for configuring visual information, thereby limiting researchers' ability to generate clear visual overviews of the AutoML workflow for their respective research communities.

Based on this constraint, we identify DR1: AutoML tools should afford researchers the ability to personalize the visual representation of AutoML workflows.

*Constraint 2—No predefined reporting*: Our findings also show that current AutoML tools have no predefined reporting features for creating text documentation. The researchers noted that AutoML tools should provide automated mechanisms to generate textual descriptions of the entire AutoML project, including information about low-level ML implementations, datasets, or hardware. One researcher explained:

“[It would be great] if I just say, I can somehow export a documentation, and then I just have a one-pager with the key parameters on it.” (P6)

While some AutoML tools offer basic features to create textual reports of e.g. hardware specifications, they still lack information about ML details such as used algorithms, hyperparameters, or optimization strategies needed to support reproducibility. Therefore, a researcher noted that AutoML tools should provide automated documentation based on templates that ensure detailed technical information:

“[There should be] a customized template which you can import, but simply that there are certain standards […] [and] that the documentation is generated automatically.” (P5)

These findings suggest that current AutoML tools lack automated documentation features that provide predefined reports of AutoML projects with comprehensive technical details.

Based on this constraint, we identify DR2: AutoML tools should afford researchers the ability to automatically create predefined reports of AutoML projects.

#### 4.1.2 Code

As LCNC AutoML tools require little to no programming code, we extend the definition of *code* to include all *executable instructions*.

*Affordance 3—Sharing workflows:* the findings from our interviews show that AutoML tools afford researchers to share workflows with other researchers using the same AutoML tools. These sharing and collaboration features can take the form of exporting entire workflows in custom file formats that can be executed by other users. One researcher experimented with this feature to share their AutoML project:

“I tried to export the model from the AutoML node into a workflow like this.” (P1)

Some AutoML tools also provide the ability to grant other users access to entire ML projects or share workflows on online platforms. A researcher explained:

“We could have shared the project […] then I would have had his data right away and could have built everything there.” (P5)

These findings suggest that AutoML tools enable researchers to share workflows either through local exports or via cloud-based access.

*Affordance 4—Recreating and executing workflows:* AutoML tools also afford researchers to recreate and execute workflows that were created by other researchers. As AutoML tools offer highly automated and standardized processes, they require less effort to implement workflows and to recreate them during reproduction. One researcher explained that these standardized AutoML processes made their reproduction attempt easier:

“Well, I think it was actually relatively easy because the structure is so similar.” (P2)

Even when AutoML workflows grow in complexity, researchers can still implement them with basic tool experience. A researcher noted that they could easily reproduce other workflows once they understood the tool:

“Given that KNIME has so many different functions […] it could be tricky to reproduce something else, but because you already understood how it was structured it was actually relatively easy.” (P2)

These findings suggest that AutoML tools offer researchers the possibility to recreate and execute workflows.

*Constraint 3—Limited workflow interoperability:* overall, researchers noted that the shared workflows of AutoML tools still have very limited workflow interoperability. Although AutoML tools offer basic workflow-sharing capabilities, imported workflows were often incompatible with other tool features or led to errors. One researcher noted that their AutoML tool recurrently failed to import workflows:

“I kept getting an error message saying that the data format was incorrect.” (P1)

Furthermore, shared workflows were often limited to the workflows and settings used in the AutoML project without including intermediate results, encountered errors, or result interpretations. A researcher explained that access to the entire original AutoML project could have helped to handle errors during reproduction:

“then I could have checked whether that [error] was the case for her too […] whether something was configured incorrectly on my end or not.” (P1)

These findings suggest that shared workflows of AutoML tools are still not interoperable with other tools and tool versions and do not allow users to configure what information is included.

Based on this constraint, we identify DR3: AutoML tools should afford researchers the ability to easily access AutoML workflows across AutoML tools and tool versions.

*Constraint 4—Missing workflow lineage:* while AutoML tools allow researchers to import and reproduce workflows developed by others, they are missing workflow lineage, including the origin of components and justifications for any applied changes. The origin of components imported from online platforms as add-ons was not clearly indicated within the tools, which made documentation difficult. One researcher explained that the tool should have made the origin of add-ons clearer:

“I think it would have been important for them to tell you to get the other nodes from the website. How are you supposed to know that?” (P4)

Researchers also struggled to determine which settings had been modified in imported workflows and why they were changed. For example, a researcher observed that default values for data splitting differed depending on the origin of certain tool components:

“If you take it from the library, I believe it uses 70% of the data to train the model […] and in the example from the KNIME-Hub that I loaded, it was set to 80%.” (P1)

These findings suggest that shared AutoML workflows currently lack essential information regarding their origin and justifications for applied configuration settings.

Based on this constraint, we identify DR4: AutoML tools should afford researchers the ability to document important design decisions of their AutoML project within the workflow.

#### 4.1.3 Data

Information about the used datasets and applied data processing procedures is essential for reproducibility.

*Affordance 5—Reporting data characteristics:* our findings indicate that AutoML tools afford researchers to effectively report data characteristics. These reports include information on dataset features, data types, and statistical analyses of outliers or missing values. One researcher explained that the tool offered a feature to simply detect outliers in the input data:

“So it was very consistent, there were hardly any outliers that I would have noticed at first glance.” (P1)

Additionally, AutoML tools can generate visualizations of these dataset characteristics, which can support comprehensive research documentation. A researcher noted that the tool automatically provided such data visualizations after the initial data upload:

“So just upload this dataset, and it will automatically recognize everything. Then you'll have a short preview, and if that's okay, you can proceed.” (P6)

These findings suggest that current AutoML tools enable researchers to analyze the characteristics of their datasets and document them in their research.

*Affordance 6—Reporting data processing*: AutoML tools also afford researchers to document data processing procedures as part of a unified ML pipeline. They provide comprehensive data processing, such as data loading, data cleaning, feature engineering, or data splitting, within their workflows. One researcher noted that they were able to easily manage, label, and split image data within their tool:

“Then there was a labeling task at the end […] [and] basically the class was divided up according to the folder structure.” (P5)

These integrated functionalities allow researchers to document and reproduce data processing steps as part of their overall ML workflow, without the need to use or reference external tools. One researcher noted that they were impressed by the included data processing capabilities of AutoML tools:

“At first, I wondered whether the tool would really be able to handle this dataset so well […] but it was actually no problem at all, so you didn't have to change anything.” (P2)

These findings suggest that the integrated features of AutoML tools allow researchers to document and reproduce data processing as part of a single ML workflow.

*Constraint 5—Missing data utility information*: while AutoML tools offer statistical information about dataset characteristics, there is missing information about data utility and risks. Researchers noted that current tools do not automatically analyze or evaluate dataset quality, which would be valuable for justifying the dataset selection in research documentation. For example, one researcher did not realize the poor data quality before implementing the entire AutoML project:

“My conclusion was that it actually works in the tool, so it's not a technical error, but it was simply a problem with the data.” (P1)

In addition, these tools lack functionality for evaluating privacy risks in their training data, which could otherwise help researchers share or anonymize sensitive information more effectively. A researcher explained that the tools should create awareness for such privacy issues:

“You don't really get any [privacy] awareness from the system, because it could include some info screens.” (P2)

These findings suggest that current AutoML tools lack information about data utility and risks that could help researchers justify their dataset choice and enable data sharing.

Based on this constraint, we identify DR5: AutoML tools should afford researchers the ability to assess data utility and risks, including concepts such as data quality, bias, and privacy.

*Constraint 6—No standardized data processing:* researchers also noted that current AutoML tools have no standardized data processing procedures, making it difficult to implement and reproduce workflows. The tools often failed to import datasets due to large file sizes or data type mismatches, without offering suggestions for resolving these errors, which resulted in several failed reproduction attempts. One researcher noted how such data import issues lead to failed reproduction attempts:

“And then I started and soon came to a standstill because I couldn't import the data.” (P4)

Moreover, some data management features only worked reliably with specific directory structures or manual adjustments to data features, making reproduction highly sensitive to user errors. A researcher explained that they were unable to recreate the original directory structure within the tool, which prevented them from reproducing the AutoML project:

“And I wasn't entirely sure how he had set up this folder structure. So, I didn't really get very far with that.” (P6)

These findings suggest that the current data processing procedures of AutoML tools lack standardization, making reproduction difficult.

Based on this constraint, we identify DR6: AutoML tools should afford researchers the ability to import and process data in a standardized way.

#### 4.1.4 Experiment

Researchers should document the details of the AutoML project, including its setup, hypotheses, results, and the computational environment with hardware and software.

*Affordance 7—Reducing stochastic variation:* our findings show that AutoML tools afford researchers the ability to reduce the stochastic variation of their results. This can be accomplished by providing random seeds in the ML pipeline. A researcher explained that they struggled to create consistent results because they did not use the tool feature for setting random seeds:

“I believe the cause was that this random seed was not set” (P1).

By enabling and documenting these random seeds, AutoML tools can enable researchers to accurately reproduce AutoML results. One researcher explained:

“In my case, every single value was the same. There was no deviation at all.” (P2)

These findings suggest that AutoML tools allow researchers to reduce the stochastic variation in their results if they set random seeds in their AutoML projects.

*Affordance 8—Reporting model performance*: furthermore, AutoML tools afford researchers to report model performance using visualizations and explainable AI (XAI) features. These tools often provide visualizations of model performance, confusion matrices, or model leaderboards that can be used for documentation. For example, one researcher used graphs of the model performance for their documentation:

“Then you can click in a little closer and view all the graphs. I did that, as required for the documentation.” (P6)

Integrated XAI features enable researchers to provide additional information, such as feature importance or saliency maps, that can enrich the documentation of research findings. One researcher documented such a saliency map for their image classification model:

“He only showed one example of this in the documentation, where he […] put a heat map over it.” (P6)

These findings suggest that AutoML tools allow researchers to report the performance of ML models using visualization and XAI features.

*Affordance 9—Reporting hardware and software setup:* AutoML tools can also afford researchers to report the hardware and software setup of their AutoML projects. The cloud AutoML tool provided a report detailing the server architecture, software versions, and overall hardware specifications used to train the models. One researcher explained that they used parts of this report to document the used server infrastructure:

“I only copied excerpts [from this report] [...] which Dataiku instance I was working on or something like that.” (P5)

The AutoML tools also provided information about software errors that appeared during training, which could be documented by the researchers alongside troubleshooting approaches. For example, a researcher failed to use the GPU for model training and documented their error handling using screenshots:

“I set up the additional GPU with five cores, and it broke down [...] I also have a screenshot [...] but then I turned it off and it worked – it's documented.” (P5)

These findings suggest that AutoML tools allow researchers to report what hardware and software they used and provide basic information about error handling.

*Constraint 7—No reproducibility guidance:* while AutoML tools provide the ability to reduce stochastic variation of results, they lack reproducibility guidance to help researchers configure the settings needed to control algorithmic randomness. Several researchers reported that the AutoML tools did not set random seeds per default, which led to different outcomes between the original implementations and the reproduction attempts. One researcher explained that the data splitting procedure could not be reproduced due to missing random seeds:

“We didn't set this random seed [...] so KNIME naturally made different decisions about which data to include in the test data and which in the training data.” (P3)

The AutoML tools also offered no reproducibility best practices that could have guided researchers in their implementations. A researcher suggested that the tool should implement pop-up windows with such guidance:

“And in the tool […] maybe you could have a pop-up reminder saying that if you want to reproduce this workflow, this point here would be individually adjustable and important.” (P4)

These findings suggest that current AutoML tools do not provide default values and user guidance to support reproducibility.

Based on this constraint, we identify DR7: AutoML tools should afford researchers the ability to understand the reproducibility implications of tool configurations.

*Constraint 8—Only expert quality metrics:* the researchers also noted that the AutoML tools provided only expert quality metrics and did not support interpretation. This included lacking explanations of performance metrics within the tools, which are often tailored to data science experts. One researcher explained:

“I think it might be better if these scores you get at the end [...] were explained more clearly in the tool.” (P2)

The tools also have no feature to compare reproduced and original model performance, which makes the interpretation of reproduction attempts harder for researchers. A researcher explained the effort required for evaluating the reproduced model performance:

“And what took the most time was really comparing the results and considering whether they were reproduced or not.” (P1)

These findings suggest that current AutoML tools provide model quality metrics that are not tailored to the needs of researchers outside data science.

Based on this constraint, we identify DR8: AutoML tools should afford researchers the ability to assess the quality of AutoML outcomes.

*Constraint 9—Unknown hardware and software interdependencies:* the researchers also reported that unclear hardware and software interdependencies complicated the reproduction attempts. Especially, the setup and configuration of the cloud AutoML tool was not intuitive. For example, one researcher noted that they were unable to install any add-ons because they lacked the necessary permissions:

“At first, I wanted to use it in the cloud. Then it was the same thing again: I couldn't install anything.” (P5)

Also, frequent software errors made reproduction difficult, especially when the tools did not provide sufficient context information. A researcher explained that they struggled to resolve software errors during reproduction attempts due to missing information:

“I think there is very little information about what the error actually is.” (P2)

These findings suggest that current AutoML tools do not provide clear information about hardware and software issues and their interdependencies.

Based on this constraint, we identify DR9: AutoML tools should afford researchers the ability to understand hardware and software interdependencies.

### 4.2 Technical review of selected LCNC AutoML tools

To evaluate the operationality of the proposed design requirements, we reviewed existing LCNC AutoML tools and identified design features corresponding to each requirement (see Table 5). The following tools were reviewed: (T1) Altair AI Studio, (T2) Orange Data Mining, (T3) KNIME, (T4) WEKA, (T5) SAS Viya, (T6) BigML, (T7) Dataiku, and (T8) DataRobot.

**Table 5:** Design features identified per design requirement and tool.

DR	Design feature	T1	T2	T3	T4	T5	T6	T7	T8
DR1	Adjustable Information	✓	✓	✓	✓	✓	–	✓	–
	Adjustable Layout	✓	–	–	✓	✓	✓	✓	✓
DR2	Automated Report Generation	–	–	–	✓	✓	✓	✓	✓
	Predefined Report Generation	–	–	–	–	✓	–	✓	–
DR3	Online workflow sharing	–	–	✓	–	✓	✓	✓	✓
	Portable workflow files	✓	✓	✓	✓	✓	✓	–	–
DR4	Workflow Descriptions	✓	–	✓	–	✓	✓	✓	✓
	Version Control System	✓	✓	✓	✓	✓	✓	✓	✓
DR5	Manual Data Assessment	✓	✓	✓	✓	✓	✓	✓	✓
	Automated Data Assessment	✓	✓	✓	–	✓	–	✓	✓
DR6	Data Collection and Import Options	✓	✓	✓	✓	✓	✓	✓	✓
	Automated Data Processing	✓	–	✓	✓	✓	-	✓	✓
DR7	Reproducibility Settings	✓	✓	–	–	–	✓	–	–
	Reproducibility Information	✓	–	✓	–	✓	✓	✓	–
DR8	Explainable AI	✓	✓	✓	✓	✓	✓	✓	✓
	Interpretation Support	–	–	✓	–	✓	✓	✓	✓
DR9	Tool Information	✓	✓	✓	✓	✓	–	✓	–
	Logging Features	–	–	–	✓	✓	✓	✓	✓

Regarding DR1, AutoML tools should enable a personalized visual representation of AutoML workflows. Existing AutoML tools provide features for adjusting the information that is available to the researchers, including features like multiple GUI views with varying levels of detail for different stages of the ML pipeline (T1, T4–6, T8). Moreover, features to show or hide advanced settings as needed (T1, T4–8), and specialized expert modes for certain ML modeling tasks that expose more technical details (T7, T8) were found. Existing AutoML tools also provide features for adjusting the layout within their GUI. This includes features like panels containing various operators or editors that can be opened, closed, or rearranged (T1–5, T7), the possibility to rearrange and align operators within the visual workflows (T1, T2, T4, T5, T7), or to create sub-workflows that can be opened and edited independently from the main workflow (T3, T5, T7). The identified design features demonstrate the operationality of DR1, as customizable GUI components can enable researchers to personalize and visually document their AutoML workflows.

Regarding DR2, AutoML tools should automatically create predefined reports of AutoML projects. AutoML tools provide features for automated report generation that generate a single standard documentation. This includes features to export information about workflows, data, or models as plain text files (T4) and to automatically generate standardized reports that provide information about every workflow step, including training settings and data lineage (T5–7). We found features for predefined report generation where the researchers can configure automatically generated documentation. This includes various preconfigured options for generating reports that explain and visualize datasets and model performance (T5) and the option to provide report templates that are automatically filled (T7). The identified design features demonstrate the operationality of DR2, as standardized reporting features can enable researchers to automatically create predefined reports of their AutoML projects that align with provided reporting guidelines.

Regarding DR3, AutoML tools need to provide easy access to AutoML workflows across AutoML tools and tool versions. To this end, some AutoML tools provide features to share workflows online with others, such as granting others access to workflows (T5, T7, T8) or datasets (T6, T7), uploading workflows to a dedicated cloud repository (T3), or publishing workflows or models as web apps (T3, T6–8). Furthermore, some AutoML tools share workflows using workflow files in standardized formats such as JSON (T3, T4, T6) or XML (T1), provide the option to export underlying code from the no-code workflows (T5), and allow sharing models and workflows as pickle files that can be loaded from Python code (T2). While such standardized file formats do not guarantee interoperability, they can make it easier to transfer or port the workflow information across different AutoML tools and versions. The identified design features demonstrate the operationality of DR3, as standardized workflow formats can enable researchers to more easily share or port AutoML workflows across AutoML tools and versions, which can aid reproduction.

Regarding DR4, AutoML tools should enable the documentation of important design decisions of an AutoML project within workflows. Several AutoML tools add workflow descriptions that further explain the components of AutoML projects. This includes features like adding text annotations to visual workflows (T1–5), adding metadata and descriptions to workflows (T2, T5, T7, T8) and datasets (T5–8), or even discussion and chat panels within workflows when cooperating in an AutoML project (T7, T8). Moreover, some AutoML tools also provide a version control system allowing researchers to track the changes of workflows and datasets (T1, T5–8) or to publish different versions of cloud workflows with comments (T3, T7, T8). The identified design features demonstrate the operationality of DR4, as features to describe and track implementation details of AutoML workflows can enable researchers to document important design decisions of their AutoML project.

Regarding DR5, AutoML tools should allow researchers to assess data utility and risks. As a basic functionality, many AutoML tools allow the manual assessment of data utility and risks focusing on data statistics for different data features or entire datasets (T1–8), visualizations of statistical correlations (T4, T6–8), or semantic information about data features and datasets (T5). Furthermore, some AutoML tools also provide features for automatically assessing data utility and risks, such as automated data quality checks for data features and datasets (T1, T3, T5, T7, T8) and the enforcement of data quality rules (T7). It also includes features for assessing data privacy by providing a dedicated risk assessment interface (T8), privacy risk estimation for every data feature (T5), or automatic data anonymization (T1, T3, T5). Also, features for data bias detection (T2, T7) and reduction (T2) were found. While data utility and risks might include other concepts apart from data quality, data bias, or data privacy, we still conclude that the identified design features demonstrate the operationality of DR5. Specifically, we argue that the integrated assessment procedures and reports for data quality, data bias, and data privacy can enable researchers to automatically assess the utility and risks associated with their datasets and thus improve data reusability.

Regarding DR6, AutoML tools should allow the import and processing of data in a standardized way. Many reviewed AutoML tools have features for collecting and importing data in a standardized way, including features for automated data collection, such as web scraping (T3) and data generation tools (T4). This also includes features for importing data in a standardized way from databases using SQL, CSV, or XML (T1–8), different cloud providers such as AWS or Snowflake (T1, T3, T5–8), or from datasets provided by the tools themselves (T1–3, T5–8). Furthermore, we found features for automated data processing that enable streamlined and standardized processes, such as automated and standardized data cleaning (T1, T3, T5, T7, T8) and automated feature engineering (T1, T4, T5, T7, T8). The identified design features demonstrate the operationality of DR6, as data processing functions present in different tools and tool versions can enable researchers to import and process data in a standardized way.

Regarding DR7, AutoML tools need to clarify the reproducibility implications of different tool configurations. AutoML tools provide dedicated reproducibility settings to reduce stochastic variation in data processing and ML model training, such as operators that use random number generation and have clear settings available to reduce stochastic variation (T1, T2, T6). Furthermore, some AutoML tools also provide detailed reproducibility information that explains the implications of different tool configurations. This includes features like tool tips that explain what setting random seeds means for the reproducibility of outcomes (T1, T3, T6, T7), informational text that warns users that random seeds might not lead to deterministic results on shared server instances (T5), or integrated AI chatbots that allow researchers to ask questions about reproducibility-related issues (T3, T7). While we acknowledge that random seeds cannot guarantee algorithmic determinism, we argue that the available features reduce stochastic variation and help researchers to better understand the reproducibility implications of different tool configurations. Thus, we argue that the identified design features demonstrate the operationality of DR7.

Regarding DR8, AutoML tools should support the quality assessment of AutoML outcomes. Current AutoML tools provide those features using explainable AI (XAI) methods. This includes typical XAI features like feature importance, saliency maps, or Shapley values (T1–3, T5–8), the possibility to access coefficients of standard statistical models (T3, T4, T6–8), or to test the effect of various input values on model predictions (T6–8). Some AutoML tools also support the interpretation of AutoML outcomes, with features such as integrated AI chatbots that help interpret performance metrics (T3, T7), explanations of performance metrics (T6, T7), and performance graphs (T5, T7) within the GUI, automated reports that explain model performance in plain language (T5), and automated detection of data leakage (T7, T8) or overfitting (T7). While XAI and interpretation support might not guarantee that researchers can understand model quality or other concepts such as predictive validity, robustness, or even successful reproduction, we consider the available features sufficient to demonstrate the operationality of DR8. Specifically, we argue that the available guidance and explanation mechanisms within the tools allow researchers without in-depth data science expertise to assess the quality of AutoML outcomes more easily.

Regarding DR9, AutoML tools should provide information about hardware and software interdependencies. Most AutoML tools provide detailed tool information, including the currently used tool version (T1–5, T7), information about the underlying server infrastructure (T5, T7), or general explanation of the tool architecture (T5). Furthermore, several AutoML tools also provide logging features that track the execution history and possible errors during execution, such as features logging the entire history of all tasks performed (T5–8), the output of all running code within a console (T4, T7), or a dedicated logging feature for every individual node in a workflow (T5, T7, T8). The identified design features demonstrate the operationality of DR9, as detailed and standardized information about the hardware and software setup within AutoML tools can enable researchers to understand interdependences and document them accordingly.

### 4.3 Design principles

To identify design principles for the development of future LCNC AutoML tools that support reproducible research, we investigated how the identified design requirements relate to their corresponding design features. Based on this investigation, we propose four design principles, following (Gregor et al. 2020), that serve as an abstraction layer between design requirements and design features (see Figure 2). We grouped design requirements and corresponding design features into a design principle when they addressed a common underlying design objective and could be addressed through related design mechanisms.

**Figure 2:**
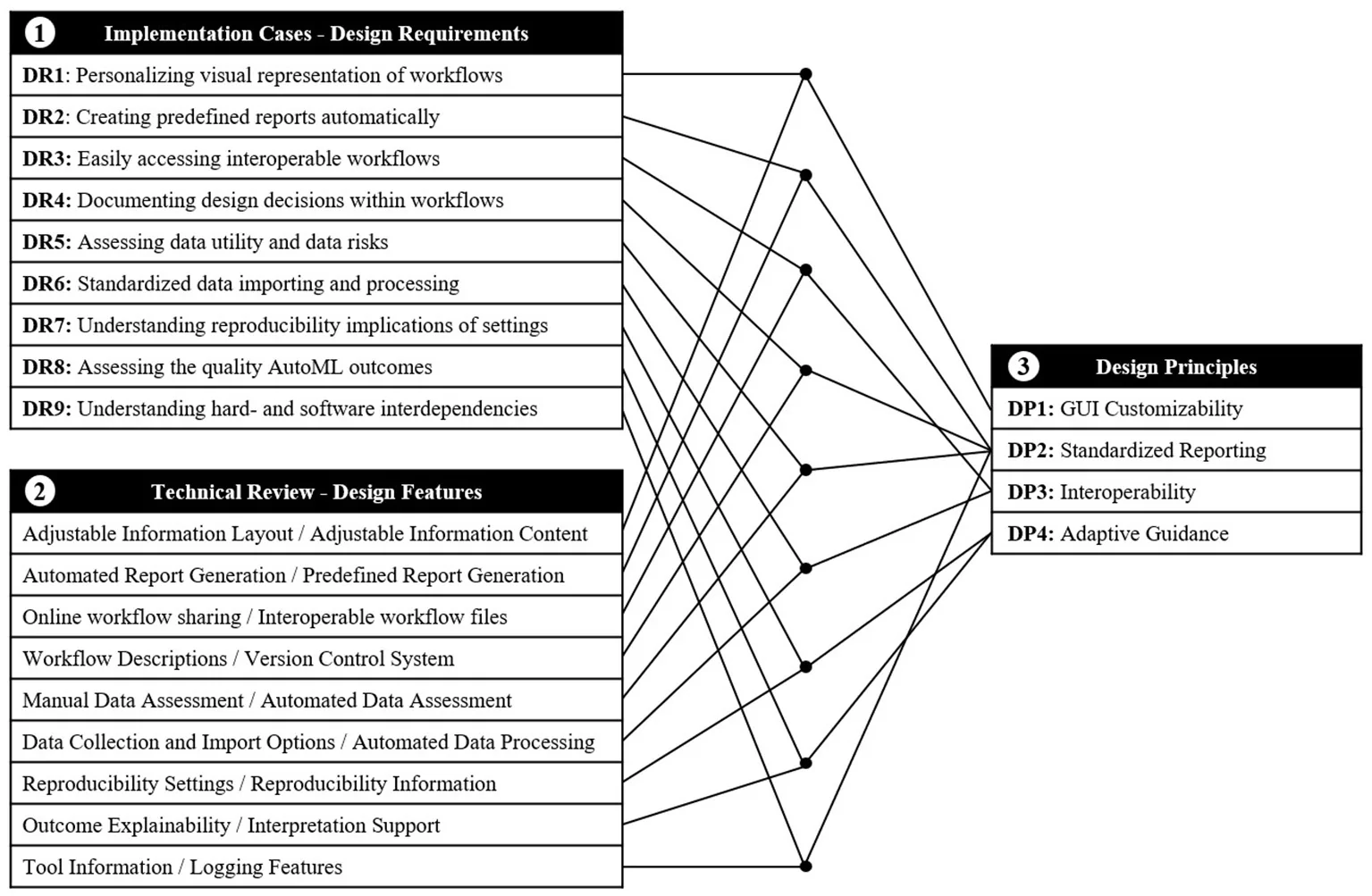
Relationships between design requirements, principles, and features.

The first design principle is *GUI Customizability*. This principle addresses DR1 that suggests that AutoML tools should afford researchers the ability to personalize the visual representation of AutoML workflows. The main design objective of DR1 is to enable custom visual documentation of research that utilizes LCNC AutoML. The corresponding design features include options to adjust the layout and information within AutoML tools. The main mechanism connecting the design requirements and design features is the customizability of GUI components that enables the personalization of visual workflow representations. This can enable researchers to adapt how they visually document their ML workflows for publications in various research domains. Therefore, we propose the following design principle:

*DP1—Principle of GUI Customizability*: to allow researchers to create personalized visual documentation, LCNC AutoML tools should provide customizable GUI components with an adjustable layout and information that fit the needs and standards of different research domains.

The second design principle is *Standardized Reporting*. This principle addresses DR2, DR4, DR5, and DR9, that call for options to automatically create reports of AutoML projects, document design decisions, assess and document data characteristics, and clarify hard- and software interdependences. The common design objective of DR2, DR4, and DR5 is to standardize and automate the documentation of AutoML workflows. The corresponding design features include report generation, workflow description, version controls, automated assessment features for data quality, bias, and privacy, as well as information about tool versions and automated logging. The main mechanism connecting the design requirements and design features is automated and standardized reporting that can be adapted to fit different documentation requirements related to data, workflow, and experimental setup. Therefore, we propose the following design principle:

*DP2—Principle of Standardized Reporting*: to allow researchers to report relevant information about data, workflows, and experimental setup, LCNC AutoML tools need to provide the option to automatically create reports that align with different documentation standards in research.

The third design principle is *Interoperability*. This principle addresses DR3 and DR6 that call for easy sharing and importing of data and access to AutoML workflows across different tools and tool versions. The common design objective of DR3 and DR6 is to simplify the recreation and execution of AutoML workflows during reproduction attempts. The corresponding design features include options to share workflows online or using dedicated workflow files and various options for standardized data import and automated data processing. The main mechanism connecting design requirements and design features is the interoperability of both ML workflows and data processing. More interoperability can ensure that ML workflows and data processing procedures can be shared and reproduced more easily across different tools and versions. Therefore, we propose the following design principle:

*DP3—Principle of Interoperability*: to allow researchers to share and import ML workflows and data processing steps, LCNC AutoML tools need to provide workflow formats and data processing procedures that are interoperable or at least portable to different AutoML tools and tool versions.

The fourth design principle is *Adaptive Guidance*. This principle addresses DR7 and DR8, which call for support mechanisms to understand the reproducibility implications of tool configurations and the quality of AutoML outcomes. The common design objective of DR7 and DR8 is to guide researchers regarding reproducible research using AutoML tools. The corresponding design features include clear settings that enable reproducible ML training, various XAI features, and different information sources within tools like chatbots. These design requirements and design features are connected by the mechanism of adaptive guidance within AutoML tools that fits the needs and requirements of researchers. This can enable researchers to understand how tool settings and their outcomes impact research reproducibility. Therefore, we propose the following design principle:

*DP4—Principle of Adaptive Guidance*: to allow researchers to understand the implications of settings and their outcomes, LCNC AutoML tools need to provide guidance mechanisms that adapt to the needs of the researchers using them.

## 5 Discussion

In the following section, we discuss the proposed design principles in relation to existing research, explore the theoretical implications of our findings, outline possible practical implications, and clarify limitations as well as directions for future research.

### 5.1 Discussion of design principles

We investigated how to design LCNC AutoML tools to support reproducible and thus responsible ML/AI research. As our main contribution, we proposed four design principles that serve as an abstraction layer between the identified design requirements and their corresponding design features.

The principle of *GUI Customizability* calls for adaptable GUI components that allow researchers to tailor the visual documentation of their AutoML workflows to the standards of different domains. The need for customizable GUI components for non-experts was already discussed in related studies (see e.g., Ardito et al., 2014; Darejeh, 2013; Xin et al., 2021). For example, (Darejeh 2013) called for a reduced number of visible features, customizable GUI elements, and avoiding expert terminology to fit the needs of users with limited IT literacy. Similarly, (Ardito et al. 2014) proposed a customizable GUI that allows non-technical users to easily manipulate visual elements to fit the information needs of different domains. For AutoML tools, (Xin et al. 2021) advocate for customizable GUIs with different modalities that hide additional settings based on user skill or intent. While these previous studies do not focus on reproducible research specifically, they align with the needs of researchers with varying levels of data science expertise to customize the GUI of AutoML tools and to visually document their workflows.

The principle of *Standardized Reporting* calls for the option in LCNC AutoML tools to automatically create reports that align with different documentation standards in research. Previous studies also highlighted the potential of automated and standardized AI documentation (Heger et al., 2022; Xin et al., 2021). (Heger et al. 2022) showed that ML practitioners want automated data documentation features that integrate into existing tools and can adapt to different industry contexts. Specifically for AutoML, (Xin et al. 2021) highlight the potential of automated and standardized documentation features that provide relevant information to support reproducibility. (Haberl and Thalmann 2026) also demonstrated that large language models (LLMs) can be used to generate standardized reports for AutoML workflows. Integrating automated documentation is especially important for AutoML users with limited data science expertise, as they are less likely to use workarounds for missing tool features (Sun et al., 2023). Overall, existing research shows the need for standardized reporting in industry or data science in general (Koenigstorfer et al., 2024b; Semmelrock et al., 2025).

The principle of *Interoperability* suggests that LCNC AutoML tools need to provide workflow formats and data processing procedures that are interoperable or at least portable to different tools and versions. The concept of interoperability is also part of the FAIR principles, which aim to improve the reusability of data (Wilkinson et al., 2016). These principles are used in data science and are incorporated in various proposed approaches for reproducible ML research (Rahrooh et al., 2024; see e.g., Shiferaw et al., 2024). However, a recent literature review by (Quaranta et al. 2025) notes that AutoML is still highly limited in its interoperability, preventing users from sharing workflows between different tools. This vendor lock-in reduces reusability and limits reproducibility in research. While our findings show the possibility of sharing open file formats (e.g., XML), the shared files might rely on semantically different schemas that still hinder interoperability (see e.g., Lampathaki et al., 2009).

The principle of *Adaptive Guidance* suggests that LCNC AutoML tools need to provide guidance mechanisms that adapt to the needs of researchers and allow them to understand the reproducibility implications of tool settings and their outcomes. While there are guidelines that support researchers in conducting reproducible ML research (see e.g., Pineau et al., 2021), they are not implemented as guidance within LCNC AutoML tools. Some approaches have emerged to help researchers understand and explain the outcomes and procedures of AutoML (Zöller et al., 2023), but they do not adapt to individual requirements. A promising avenue is the use of LLMs that guide users with varying levels of ML expertise through the AutoML workflow (Bakas et al., 2025) or help users interpret AutoML outcomes (Gigerl et al., 2026). Overall, LLMs emerge as promising solutions for adaptive guidance in many domains (see e.g., Kheirabadi et al., 2025; Song et al., 2025).

### 5.2 Theoretical implications

These design principles can serve as actionable design guidance for developing LCNC AutoML tools that address reproducibility challenges in research. One major issue discussed in the AutoML literature is the lack of transparency in underlying AutoML implementations (Sun et al., 2023; Xin et al., 2021), which hinders researchers' ability to document their workflows. Participants in our study particularly struggled to access information about hardware and software interdependencies. Implementing standardized reporting mechanisms and customizable GUIs that allow users to adjust information could therefore enhance documentation quality and reproducibility. Another challenge is that sharing code like in traditional ML research (Semmelrock et al., 2025) is not possible in many LCNC AutoML platforms, complicating the reuse of workflows (Haberl and Thalmann, 2025). To promote workflow reusability, it is essential to develop interoperable workflow formats that are compatible or portable across different AutoML tools. A further challenge comes from the limited ML expertise of users, which is often associated with lower awareness of reproducibility (Pletzl et al., 2024) and data privacy concerns (Sun et al., 2023). Our findings indicate that implementing adaptive guidance mechanisms that assist these researchers is crucial.

Our findings also demonstrated that TACT (Majchrzak and Markus, 2012) can serve as a valuable theoretical lens in the context of design science research. While standard qualitative data analysis methods already allow researchers to identify themes from underlying material (see e.g., Mayring, 2014), using TACT as a guiding framework can align those themes more closely to the interaction of users with specific technology. This relational perspective of affordance-based theories (see e.g., Bernhard et al., 2013; Mesgari et al., 2023) seems to be especially informative when identifying design knowledge for future technologies (see e.g., Klemm and Reintjes, 2026; Li et al., 2026). In our study, this perspective allowed us to identify design principles that address constraints emerging when researchers with limited ML expertise interact with AutoML tools. For example, while current tools allow researchers to set random seeds, they constrain users by offering little guidance on how this affects reproducibility. By analyzing these affordances and constraints, we formulated DR 7, which states that tools should enable researchers to understand the reproducibility implications of their chosen settings. From that followed DP 4, which proposes adaptive guidance in future tools to help researchers understand those implications. Similarly, the principles of GUI customizability, standardized reporting, and interoperability specifically suggest how tool design can ultimately reduce constraints and provide reproducibility-related affordances for researchers.

### 5.3 Practical implications

The findings of this design science study also have practical implications for tool developers and researchers alike. As many AutoML tools are currently utilized by industry users (see e.g., Scriven et al., 2023; Sundberg and Holmström, 2023), they often do not provide reproducible processes out of the box (Gundersen et al., 2022). By incorporating the design principles identified in this study, tool developers can meet the needs of both industry practitioners and researchers. While reproducibility is often not the main focus in industry, providing explainability and transparency across the entire lifecycle of ML operations is also relevant for quality assurance and regulatory compliance (Moosavi et al., 2024; Tekkesinoglu et al., 2026). In this regard, incorporating our design principles into LCNC AutoML tools can also help companies to ensure transparent ML operations, especially in regulated industries where specific AI documentation is needed (Königstorfer and Thalmann, 2021).

As AutoML tools often lack customizability to fit the needs of different domains (Sun et al., 2023), the design principles of this study can help independent developers create extensions of open-source AutoML tools (see e.g., Fillbrunn et al., 2017), thereby strengthening the overall reproducibility of research in various domains. Specifically, incorporating GUI customizability and standardized reporting can help researchers adapt and automate domain-specific research documentation. Improving interoperability can aid effective workflow and data sharing and enable easier reproduction of research findings. Finally, adaptive guidance can help researchers better understand the outcomes and implications of tool settings, thereby enabling them to create comprehensive research documentation. In this way, this study also contributes to more transparent and responsible research practices that enhance the trustworthiness of scientific findings.

### 5.4 Limitations and future work

This study has some limitations. Although the participating researchers had diverse backgrounds, several fields, particularly within the life and natural sciences, were underrepresented. Future research could involve a broader set of disciplines with varying documentation standards to strengthen the robustness of the identified design knowledge. We also relied on the researchers' main research domain as a proxy for their expertise in data science. Thus, future research should ensure to measure AI literacy (see e.g., Laupichler et al., 2023) or tool experience of participants explicitly to ensure the validity of findings associated with the use of LCNC AutoML tools by non-experts. Furthermore, the researchers participating in this study were all located at a single Austrian university, which risks biases based on institutional culture and academic socialization. Thus, future studies should aim to include participants from multiple research institutions and with more diverse academic socialization backgrounds. Similarly, many widely used LCNC AutoML tools were investigated in the technical review and used for the implementation cases, but numerous others were not examined. Furthermore, the implementation cases were also created with only two different LCNC AutoML tools. Future studies could therefore assess the applicability of our findings to a wider range of tools. Moreover, while our design principles were based on empirical findings and informed by insights from the literature and existing tools, they were not instantiated in concrete design artifacts. Subsequent research should evaluate prototype implementations in real research settings to determine their impact on documentation quality and reproducibility.

## 6 Conclusion

This design science study explored how to design LCNC AutoML tools that can support reproducible research. To this end, we contributed design knowledge in the form of identified design requirements, corresponding design features, and resulting design principles. Using the theoretical lens of TACT, we identified design requirements through the analysis of implementation cases and interviews with researchers. To evaluate the operationality of these requirements, we conducted a technical review of existing LCNC AutoML tools to identify corresponding design features. From this analysis, we formulated four design principles (GUI Customizability, Standardized Reporting, Interoperability, and Adaptive Guidance). The four design principles provide a basis for designing LCNC AutoML tools that better support the documentation and reproducibility of ML research. Future work should instantiate and empirically evaluate these principles in real research settings.

## Data Availability

The original contributions presented in the study are included in the article/Supplementary material, further inquiries can be directed to the corresponding author.

## Footnotes

1
https://www.knime.com/

2
https://www.dataiku.com/
