# Supplementary material for: Reproducible research by design: design principles for low-code/no-code AutoML tools
Source: Front Res Metr Anal. 2026 Oct 16;11:1967319. doi: 10.3389/frma.2026.1967319 (PMC13719566; doi:10.3389/frma.2026.1967319)
Supplement: Supplementary file 1 [file Data_Sheet_1.docx]

# Codebook

| **Category** | **Theme** | **Description** | **Exemplary Quote** |
| --- | --- | --- | --- |
| **Description** | Affordance - Creating visual descriptions | This theme subsumes codes related to using AutoML tool GUIs to document project workflows, configurations, and results via screenshots and video recordings. | *“People need a video showing what it looks like in the workflow, because it's hard to describe on paper. I took extremely many screenshots […] And in a video, that's just enough.”* |
|  | Affordance - Creating individual reports | This theme subsumes codes related to how the streamlined workflows and limited configuration options of AutoML tools facilitate simple, concise, and manageable individual project documentation. | *“So, I basically just tried to write down everything I did in the tool as precisely as possible, creating a click-by-click guide, so to speak, so that it's transparent how I arrived at the result.”* |
|  | Constraint - Fixed visual information | This theme subsumes codes related to how fixed, opaque, and highly technical visual information in AutoML tools hinders documentation of underlying workflows, implementation details, and automated decisions. | *“The AutoML node doesn't actually export a model, but rather an entire separate workflow is behind it […] but I don't know how I could have figured that out on my own.”* |
|  | Constraint - No predefined reporting | This theme subsumes codes related to the current limitations, time-consuming nature, and specific feature requirements for automated and template-based project documentation in AutoML tools to support reproducibility and workflow tracking. | *"And then maybe you deploy it, it's in production, but actually you might need documentation and then you write it after the fact because there is so much work involved."* |
| **Code** | Affordance - Sharing workflows | This theme subsumes codes related to how AutoML tools enable researchers to collaborate and share machine learning workflows, projects, and models through local file exports or cloud-based user access. | *"Very originally, there was an attempt to also work with his uploaded data, because we jointly [have access to] this Dataiku Hub."* |
|  | Affordance - Recreating and executing workflows | This theme subsumes codes related to the factors, processes, and tool features, such as standardization, workflow complexity, and prior tool experience, that influence the ease or difficulty of recreating and executing AutoML workflows. | *"And exactly, then I actually had no problems at all with this reproduction and also with understanding this result, because everything was the same."* |
|  | Constraint - Limited workflow interoperability | This theme subsumes codes related to the technical limitations and errors encountered when sharing, exporting, and reproducing AutoML workflows and full machine learning projects across different tools or user environments. | *"Even if I... selected Gradient-Boosted Trees or something in the AutoML tool and then took a Gradient-Boosted Trees Executor or something like that, it just didn't fit together."* |
|  | Constraint - Missing workflow lineage | This theme subsumes codes related to the difficulty of tracing and documenting the lineage of shared AutoML workflows, specifically regarding the unclear provenance of downloaded add-ons and undocumented configuration differences between default tool settings and online templates. | *"I think I left the default settings everywhere, but I just noticed that they were different, or partly different, in the finished model from the documentation than when you take them directly from the catalog."* |

| **Data** | Affordance - Reporting data characteristics | This theme subsumes codes related to how AutoML tools automatically describe, visualize, and identify data features, such as missing values, outliers, and data types, to assist researchers in reporting dataset characteristics. | *"There was no missing data at all, everything was filled in."* |
| --- | --- | --- | --- |
|  | Affordance - Reporting data processing | This theme subsumes codes related to how integrated AutoML functionalities facilitate the end-to-end execution, simplified management, and documentation of data processing tasks, including data loading, labeling, feature engineering, and splitting. | *"And there it made the assumption itself, and I have to say, I hadn't even thought about that for my dataset, for example. And it was actually really important to make that step."* |
|  | Constraint - Missing data utility information | This theme subsumes codes related to the limitations of AutoML tools in automatically analyzing data quality and evaluating legal, privacy, or ethical risks, which restricts researchers' ability to justify dataset selection and ensure secure data handling. | *"For example, if you naturally leave some sensitive data in or if you... so things like that, so naturally data privacy is always, I think, the sticking point [with these tools]"* |
|  | Constraint - No standardized data processing | This theme subsumes codes related to the fragile and unstandardized nature of data processing in AutoML tools, specifically challenges in data import, management, and preparation that hinder workflow reproducibility. | *"Data import was just very complicated. If you can't get the data in, you can't build an AutoML either."* |
| **Experiment** | Affordance - Reducing stochastic variation | This theme subsumes codes related to how AutoML tools enable the generation of results with low stochastic variation, primarily through setting random seeds across pipeline steps like data splitting and model training, as well as replicating prior machine learning findings. | *"And then I immediately started to rebuild it, and I think it wasn't even saved once and I did it again, so I reproduced it twice and it still always came out the same."* |
|  | Affordance - Reporting model performance | This theme subsumes codes related to how AutoML tools support the documentation and interpretation of machine learning results through automated performance visualizations, metrics, and explainability (XAI) features. | *"For each of these models, there are these parameters that you provided, and then there's always a number listed next to them […] and in the end, it shows that the model performed this much better."* |
|  | Affordance - Reporting hardware and software setup | This theme subsumes codes related to how AutoML tools provide system information and logs that enable researchers to document their computational environment, training duration, and error troubleshooting. | *"And then, with the first one it said, GPU-Usage was requested for Computer Vision, but no GPU is available."* |
|  | Constraint - No reproducibility guidance | This theme subsumes codes related to the lack of default deterministic settings like missing random seeds, and missing user guidance within AutoML tools that cause non-reproducible model training, data splitting, and performance results. | *"If we had said beforehand, okay, well, the random seed has to be set […] then comparability could have been evaluated better from a reproducibility standpoint.”* |
|  | Constraint - Only expert quality metrics | This theme subsumes codes related to the lack of built-in support, explanations, and comparative features in AutoML tools for interpreting performance metrics and evaluating reproduction success, particularly for non-data science experts. | *"So, I think that the interpretation of these metrics, that just remains a bit difficult for the non-IT expert."* |
|  | Constraint - Unknown hardware and software interdependencies | This theme subsumes codes related to complex hardware and software interdependencies, technical complications, setup hurdles, and opaque error messages in cloud AutoML tools that hinder reproduction efforts. | *"The one thing, that there were simply error messages where I didn't know at all [what to do]."* |
