# Supplementary material for: Reproducible research by design: design principles for low-code/no-code AutoML tools
Source: Front Res Metr Anal. 2026 Oct 16;11:1967319. doi: 10.3389/frma.2026.1967319 (PMC13719566; doi:10.3389/frma.2026.1967319)
Supplement: Supplementary file 2 [file Data_Sheet_2.docx]

Documentation of the Implementation

# 1. Research Context and Research Question

## 1.1. Research Context

Describe the broader research context in which your use case is embedded. This should include the scientific field and the relevance of the project.

## 1.2. Research Question

Formulate the specific research question you aim to answer with your AutoML project. This question should be precise and measurable.

# 2. Dataset Used and Data Preparation

## 2.1. Dataset Description

Describe the dataset used, its source, and its key characteristics (e.g., number of instances, number of features, class distribution, etc.). Include a link or the exact origin of the dataset if it is publicly accessible.

## 2.2. Data Preparation

Describe the data cleaning and preparation steps. What type of preprocessing was performed before loading the dataset into the AutoML tool? Address topics such as missing values, feature engineering, normalization, or categorization.

# 3. Methodology

## 3.1. AutoML Tool Used

Describe the AutoML tool you utilized. State the name and version of the software (e.g., Google AutoML, H2O.ai, KNIME, etc.). Briefly explain why you chose this tool.

## 3.2. Hardware Setup

Specify the hardware specs used to execute the AutoML workflow. This includes details on the operating system, CPU, GPU, and RAM capacity. Also specify whether the training was performed locally on your PC or in the cloud.

## 3.3. Workflow in the Tool

Explain step-by-step how the model was created in the AutoML tool. This should include parameter selection, model types (if manually selectable), training settings, duration of model training, and any manual interventions. Document how the model's performance was evaluated as well. You may also include screenshots or videos of the tool in use.

# 4. Results

## 4.1. Performance Evaluation

Present the key performance metrics of the model (F1-score, Precision, Recall, ROC-AUC, etc.). Ensure the results are clearly presented, e.g., in tabular format.

## 4.2. Model Interpretation

If relevant, provide information on model explainability, such as feature importance, Shapley values, or other methods offered by the AutoML tool to explain predictions.

# 5. Appendix

## 5.1. Source Code and Configuration Files

If relevant, attach all scripts, configuration files, and settings used for data preparation, model selection, or evaluation.

## 5.2. Screenshots of Tool Usage

Document the key steps using screenshots, especially if manual adjustments were made in the AutoML tool that are essential for reproducibility.

## 5.3. Link to Dataset and Model Deployment (if publicly accessible)

If the model or dataset is publicly accessible, provide the corresponding links.
