# Supplementary material for: Reproducible research by design: design principles for low-code/no-code AutoML tools
Source: Front Res Metr Anal. 2026 Oct 16;11:1967319. doi: 10.3389/frma.2026.1967319 (PMC13719566; doi:10.3389/frma.2026.1967319)
Supplement: Supplementary file 3 [file Data_Sheet_3.docx]

Documentation of Reproduction

# 1. Perspectives and Success of Reproduction

The reproducibility of scientific results can be assessed from different perspectives. Goodman et al. (2016) distinguish between three types of reproducibility:

- Methods Reproducibility: The methodological procedure can be repeated.
- Results Reproducibility: The same results can be achieved again.
- Inferential Reproducibility: The same interpretation of the results is possible.

In the context of this study, you should attempt to reproduce the AutoML project of your tandem partner while considering all three perspectives of reproducibility.

## 1.1. Methods

Describe the extent to which you were able to understand and reproduce the methodological procedure of your tandem partner.

## 1.2. Results

Describe which performance metrics of the AutoML project you intend to reproduce. Specifically address quantitative metrics such as Precision, R², RMSE, etc., documented by your tandem partner. Additionally, define the tolerance thresholds required for a successful reproduction (e.g., ±5%). To what extent were you able to reproduce the defined performance metrics?

## 1.3. Interpretation

Describe the extent to which you were able to understand and reproduce your tandem partner's interpretation of the results. You may refer to both the interpretations in the documentation and your tandem partner's presentation.

# 2. Procedure

In the first step, you should attempt to reproduce your tandem partner's AutoML project without their help. If you encounter difficulties or have questions, you may subsequently contact your tandem partner. Document which steps you were able to complete independently, what information you required from your tandem partner, and in which aspects you deviated from your tandem partner's original approach.

## 2.1. Independent Reproduction

Describe your approach to independently reproducing the AutoML project. Which steps, results, and interpretations were you able to reproduce independently? What problems occurred during the process? Also address any differences that arose between the initial reproduction attempt and subsequent attempts.

## 2.2. Interaction with the Tandem Partner

Describe what information or support you received from your tandem partner in order to reproduce the AutoML project. How could the documentation have been improved to facilitate independent reproduction?

## 2.3. Deviations from the Tandem Partner

Describe in which aspects you deviated from your tandem partner's procedure. Justify why these deviations were necessary to enable a successful reproduction of the results and the interpretation.
