# Supplementary material for: Reproducible research by design: design principles for low-code/no-code AutoML tools
Source: Front Res Metr Anal. 2026 Oct 16;11:1967319. doi: 10.3389/frma.2026.1967319 (PMC13719566; doi:10.3389/frma.2026.1967319)
Supplement: Supplementary file 4 [file Data_Sheet_4.docx]

Detailed Overview of Implementation Cases

| **Tool** | **Researcher Domain** | **Project Description** | **ML Task** | **Data type and  Link to Dataset** | **Target Variable** | **Initial Outcome** | **Reproduction Outcome** | **Discrepancies and  likely causes** |
| --- | --- | --- | --- | --- | --- | --- | --- | --- |
| KNIME | Psychology | The project aimed at predicting the stress levels of employees based on personal and work-related variables. | Classification | Tabular Data  <https://www.kaggle.com/datasets/waqi786/remote-work-and-mental-health> | Stress_Level (ordinal feature) | Gradient-Boosted-Tree Model with overall accuracy of 0.3525 | Gradient-Boosted-Tree Model with overall accuracy of 0.3525 | No discrepancies,  100% identical results;  Random seeds were set during data splitting and model training |
| KNIME | Law | The project was about a personal interest of the researcher and aimed at predicting the powder performance of different snowboards. | Classification | Tabular Data  <https://www.kaggle.com/datasets/oznbgcvn/snowboardpowdervalue> | Powder  (ordinal feature) | Random Forest Model with F1-Scores ranging from 0.182 to 0.774 for the different powder levels | Random Forest Model with F1-Scores ranging from 0.286 to 0.833 for the different powder levels | Noticeable performance differences, likely due to missing random seed (data splitting, model training) and limited dataset size |
| KNIME | Business | The project aimed at predicting future sales of a retail store based on data including seasonal and product related features. | Regression | Tabular Data  <https://www.kaggle.com/datasets/abdullah0a/retail-sales-data-with-seasonal-trends-and-marketing> | RETAIL SALES (numerical feature) | Linear Regression Model with R^2 of 0.887 | Linear Regression Model with R^2 of 0.887 | No discrepancies,  100% identical results,  However, reproduction was time-consuming due to undocumented errors during data import |
| KNIME | Sociology | The project aimed at predicting health insurance charges based on demographic data and basic health and lifestyle indicators. | Regression | Tabular Data  <https://www.kaggle.com/datasets/willianoliveiragibin/healthcare-insurance> | Charges (numerical feature) | Regression Model with R^2 of 0.853 | Regression Model with R^2 of 0.836 | Slight performance differences, likely due to missing random seeds during data splitting and model training; Reproduction difficult due to minimalistic documentation of project |
| Dataiku | Business | The project was about a personal interest of the researcher and aimed at classifying X-Ray images for pneumonia detection. | Classification | Image Data  <https://www.kaggle.com/datasets/paultimothymooney/chest-xray-pneumonia> | Label  (nominal feature) | Neural Network with F1-Score of 0.89 | Neural Network with F1-Score of 0.86 | Slight performance differences, likely due to missing random seeds; Reproduction was difficult due to persisting errors during data import |
| Dataiku | Ethics | The project was about predicting the attrition of employees based on employee characteristics. | Classification | Tabular Data  <https://www.kaggle.com/datasets/pavansubhasht/ibm-hr-analytics-attrition-dataset> | Attrition (binary feature) | Logistic Regression with ROC AUC of 0.851 | Logistic Regression with ROC AUC of 0.851 | No discrepancies,  100% identical results, Tool set random seeds by default, Documentation included video instructions which made reproduction simple |
