# Supplementary material for: Reproducible research by design: design principles for low-code/no-code AutoML tools
Source: Front Res Metr Anal. 2026 Oct 16;11:1967319. doi: 10.3389/frma.2026.1967319 (PMC13719566; doi:10.3389/frma.2026.1967319)
Supplement: Supplementary file 5 [file Data_Sheet_5.docx]

Interview Guideline: Implementation

# Initial Situation

- What research goal did you want to achieve as part of this AutoML project, and why did you choose this goal?
- How would you describe your domain-specific prior knowledge regarding this research goal?
- How familiar are you generally with IT, data preparation methods, or quantitative research methods?
- How familiar were you with Machine Learning or AutoML before you started this project?

# Onboarding and Familiarization

- How did you familiarize yourself with your chosen AutoML tool at the beginning of the project?
- How did you familiarize yourself with the chosen dataset, and how did you prepare it?
- What resources (e.g., videos, forums, etc.) did you use during onboarding, and how did they help you?
- What difficulties did you face during this onboarding phase, and how did you handle them?
- What additional information or tool features would you have needed to familiarize yourself better with the tool?

# Model Creation and Evaluation

- What work steps did you take (and how often) to create your ML model?
- How did you evaluate the quality of your model, and which criteria were decisive for this?
- Which tool features did you use, and why did you choose them?
- What difficulties did you encounter when creating and evaluating your ML model, and how did you handle them?
- What additional information or tool features would you have needed to better create and evaluate the ML model?

# Documentation

- How did you document your AutoML project so that it can subsequently be reproduced by other participants?
- When did you create the documentation - during the creation of the model or afterward?
- Which resources or tool features were particularly helpful for creating your documentation?
- Apart from the aspects in the provided documentation template, what other aspects should be considered during documentation?
- What difficulties did you encounter when documenting your AutoML implementation, and how did you handle them?
- What additional information or tool features would you have needed to better document your AutoML project?

# Learning Effects

- What key takeaways can you draw from this AutoML project for yourself and your research?
- What did you learn about your field of research or the topic of your research project during the implementation of your AutoML project?
- To what extent did using the AutoML tool help you gain a better understanding of machine learning and AI?
- Did the experience from this project prepare you well for potential future AutoML projects? How confident do you feel with AutoML?
- Which resources or tool features were particularly educational for you, and how could they be improved?

# Conclusion

- To what extent were you able to achieve the research goal you set for yourself in this AutoML project?
- What is your overall impression of AutoML tools and their application in research?
- For which future research activities could AutoML be helpful to you?
- What problems and challenges do you see when using AutoML in research, and how could they be addressed?
- Do you have any additional feedback?
