# Supplementary material for: Reproducible research by design: design principles for low-code/no-code AutoML tools
Source: Front Res Metr Anal. 2026 Oct 16;11:1967319. doi: 10.3389/frma.2026.1967319 (PMC13719566; doi:10.3389/frma.2026.1967319)
Supplement: Supplementary file 6 [file Data_Sheet_6.docx]

Interview Guideline: Reproduction

# Initial Situation

- How would you describe your tandem partner's AutoML project in terms of topic, data, and results?
- What was your impression of your tandem partner's implementation, what surprised you, and what would you have done differently?
- How did the communication with your tandem partner work during the reproduction phase, and how could it have worked better?

# Reproduction

- What were your criteria for assessing the reproducibility of your tandem partner's AutoML project?
- What steps did you take to verify the reproducibility of your tandem partner's AutoML project?
- Which features of the AutoML tool were particularly helpful during reproduction, and where do you see room for improvement?
- How would you describe the quality of your tandem partner's documentation, and where do you see room for improvement?
- What additional information (e.g., guidelines, videos, etc.) did you use, or would have potentially been helpful?
- Which steps in reproducing your tandem partner's AutoML project were particularly effort-intensive or difficult for you?
- To what extent were you able to reproduce your tandem partner's AutoML project?

# Conclusion and Recommendations

- What challenges do you see regarding reproducibility when using AutoML in research?
- From your perspective, what are possible approaches to support the reproducibility of AutoML research?
- What recommendations do you have for the responsible use of AutoML in research?
- Do you have any additional feedback?
